# Edible bird’s nest impact on rats’ uterine histomorphology, expressions of genes of growth factors and proliferating cell nuclear antigen, and oxidative stress level

**DOI:** 10.14202/vetworld.2018.71-79

**Published:** 2018-01-27

**Authors:** Abdulla A. Albishtue, Nurhusien Yimer, Md Zuki A. Zakaria, Abd Wahid Haron, Rosnina Yusoff, Mohammed A. Assi, Bahaa H. Almhanawi

**Affiliations:** 1 Department of Veterinary Clinical Studies, Faculty of Veterinary Medicine, Universiti Putra Malaysia, 43400 Serdang, Selangor, Malaysia; 2 Department of Anatomy and Histology, Faculty of Veterinary Medicine, University of Kufa, Najaf, Iraq; 3 Department of Veterinary Preclinical Sciences, Faculty of Veterinary Medicine, Universiti Putra Malaysia, 43400 Serdang, Selangor, Malaysia; 4 Department of Community Health, College of Health and Medical Techniques, Al-Furat Al-Awsat Technical University, Iraq; 5 Department of Pathology, Faculty of Medicine and Health Sciences, Universiti Putra Malaysia, 43400 Serdang, Selangor, Malaysia

**Keywords:** antioxidant, edible bird’s nest, growth factors, proliferating cell nuclear antigen, uterus

## Abstract

**Aim:**

This study aimed to evaluate the effect of edible bird’s nest (EBN) supplementation on the uteri of rats based on analyses of the morphological and histomorphometric changes, and expressions of epidermal growth factor (EGF) and its receptor (REGF) genes, vascular endothelial growth factor (VEGF), proliferating cell nuclear antigen (PCNA), and steroid receptors.

**Materials and Methods:**

Twenty-four: Sprague Dawley rats were equally distributed into the following four groups: G1 (control), G2, G3, and G4 represented the groups treated with EBN at graded concentrations of 0, 30, 60, and 120 mg/kg body weight (BW) per day for 8 weeks, respectively. During the experimental period, the BW of each rat was recorded weekly. At the proestrus stage of estrous cycle, blood samples were collected from the hearts of anesthetized rats that were later sacrificed. The uteri were removed for histological and immunohistochemical analyses.

**Results:**

The EBN-treated groups showed an increase in the weights and lengths of uteri as compared to the control. Results showed that relative to G1 and G2, G3 and G4 exhibited proliferation in their uterine luminal and glandular epithelia and uterine glands, and up-regulated expressions of EGF, REGF, VEGF, PCNA, and progesterone receptor, and estrogen receptor in their uteri. The EBN increased the antioxidant (AO) and total AO capacities and reduced the oxidative stress (OS) levels in non-pregnant rats.

**Conclusion:**

Findings of this study revealed that EBN promotes proliferation of the uterine structures as evidenced by the upregulation of the expressions of steroid receptors, EGF, REGF, VEGF, and PCNA in the uterus and increased in the plasma concentrations of AO and reduced levels of OS.

## Introduction

The female reproductive tract including the uterus, ovaries, and mammary glands is all the targets of estrogen and progesterone actions [1]. The uterine effects of estrogen and progesterone are primarily executed by nuclear estrogen receptor (E_2_R) and progesterone receptor (P_4_R) [2]. Progesterone is essential for implantation and pregnancy maintenance in all mammals and plays a role in the proliferation, differentiation, and maintenance of endometrial stromal, glandular, and myometrial cells. Estrogen is vital for the proliferation of uterine epithelia and enhances the progesterone action by inducing the PR gene. Low estrogen levels in women have been associated with elevated rates of menstrual dysfunction, such as amenorrhea and irregular menstruation [3]. A study on cognitive aging by researchers from Universiti Putra Malaysia (UPM) has recently reported an increased serum estradiol level found in ovariectomized rats fed with edible bird’s nest (EBN) dietary supplement. Hence, the scholars concluded that EBN can be used as estrogen therapy for ovariectomized-induced, aging-related memory loss [4]. Such work reinforced the findings of previous studies that EBN contains estradiol. Matsukawa *et al*. [5] studied the effect of dietary EBN extract against osteoporosis in ovariectomized rats and also observed that the serum estradiol levels in ovariectomized rats were similar to those in non-ovariectomized rats. This result reflected the compensatory role of the EBN extract for estrogen (E_2_) in the absence of the ovary and may benefit postmenopausal women with high osteoporosis risk. The EBN possesses other biological properties that may potentially influence mammalian reproduction and fertility. These properties include its high sialic acid content [6], epidermal growth factor (EGF)-like activity [7], and a stimulatory effect on cell growth and regeneration [8,9]. Emerging research findings on EBN in areas other than reproduction have shown that EBN enhances the production of reproductive hormones, such as estrogen, and serves as an antioxidant (AO). Sialic acid constitutes the greatest proportion of sugar in EBN and is an important molecule found in all animal cells. This compound plays several biological roles, such as in cell communication and as a signaling molecule [10]. Sen *et al*. [11] described the estradiol-mediated regulating role of sialic acid in the binding protein expression in human endometrium.

The present study aimed to investigate the role of *in vivo* supplementation of EBN in female reproduction using a rat model. We fed EBN extracts to rats at different concentrations, conducted gross and histomorphometric analyses of reproductive tissues, and determined the reproductive parameters and associated biomarkers. We also detected the expression of genes and their receptors (EGF and receptor of epidermal growth factor [REGF], vascular endothelial growth factor [VEGF], and proliferating cell nuclear antigen [PCNA]) and assessed their degree of protection against oxidative stress (OS) damage by standard techniques.

## Materials and Methods

### Ethical approval

All procedures performed according to Institutional Animal Care and Use Committee guideline, UPM (Project approval number: UPM/IACUC/AUPR009/2016).

### EBN preparation

EBN was purchased from Nest Excel Resources Sdn Bhd and maintained at 25°C-30°C. EBN extract was prepared in accordance with the Chinese tradition as indicated by the local EBN suppliers. The samples were cleaned, dried at room temperature, and ground into powder using a mixer (BUCHI-400, Switzerland). The ground EBN extract was maintained at 4°C. EBN solution was prepared by dissolving 1 g of EBN powder in 100 mL of distilled water (DW), followed by heating in a water bath at 60°C for 45 min. Finally, the EBN solution was cooled down to room temperature and administered to the rats at doses based on their body weights (BWs).

### Animals and experimental design

This study embodied a 24 (6×4 groups) adult female Sprague Dawley rats from the Animal Resource Center, Faculty of Veterinary Medicine, UPM. The rats were housed in cages in groups for an acclimation period of 7 days with free access to water and standard rat diet (Gold Coin Brand Animal Feed, Malaysia). After 7 days period of acclimation, the animals were categorized into four groups and supplemented with EBN solution on the basis of Table-1 for 8 weeks. All the groups were under free access to water. The determination of EBN dose was based on a previous report as described by Ismail *et al*. [4]. During the administration period, the rat BWs in each group were measured and recorded every week. At the proestrus stage, the rats were euthanized at stipulated dates (8 weeks) by CO_2_ asphyxiation method following a general anesthesia procedure, which included administration of 30 mg ketamine/kg BW and 10 mg xylazine/kg BW for blood collection.

**Table 1:** Animal grouping and treatment regime of EBN administered by gavage needle.

Group	Group assigned	Type of feed (dose)
Control	G1 (No dose)	ND+Normal saline (1 mL)
Treated	G2 (Low dose)	ND+EBN (30 mg/kg body weight)
	G3 (Medium dose)	ND+EBN (60 mg/kg body weight)
	G4 (High dose)	ND+EBN (120 mg/kg body weight)

EBN=Edible bird’s nest, ND=Normal diet

### Determination of estrous cycle phases and synchronization

Every day during the experimental period, vaginal smears were obtained in the morning at 09:00-10:00 h using wooden swab sticks with cotton tips. The tips were then moistened by dipping into the distilled water and then inserted carefully into the vagina to about 1.0 cm. The swab was subsequently smeared onto a sterile microscopic slide (75 mm×25 mm) through a rotatory movement of the tip. The slide was fixed with methanol and stained with 5% Giemsa stain for 3 min and then observed under a light microscope (Leitz Laborlux-S, Wetzlar, Germany) with 40× objective lenses to determine the percentage of cells in the vaginal smear. The rats were synchronized for estrus using two intraperitoneal doses of 0.5 mg of cloprostenol 3 days apart [12].

### Macroscopic and microscopic examinations of the reproductive tract and liver

After sacrificing the anesthetized rats, their liver and uterus were excised, weighed, and measured in length. Any evident abnormality found during gross examination was recorded. The histology samples were fixed in 10% formalin for 24 h, sectioned, and stained using hematoxylin and eosin (H & E). The samples were observed under a microscope for histological changes, including those in the endothelial lining and the number of uterine glands [13]. Sections were cut from the central region to the peripheral region. The thicknesses of the luminal epithelium (LE), glandular epithelium (GE), and endothelium, and the number of uterine glands were determined using the Medical Image Analysis software (Motic Image plus 2.0, China).

### Analyses of the expression of EGF, REGF, PCNA, VEGF, E_2_R, and P_4_R in uterine tissues by immunohistochemistry

The protein expression was investigated by immunohistochemistry. Samples were sliced (4 µm thickness), deparaffinized, and dehydrated. Antigens were retrieved by incubating the specimens in 0.01 M citrate buffer (pH 6.0) at 80°C for 10 min, cooling naturally to RT. Specimens were rinsed for 1 min in Tris-buffered saline (TBS), marked with a Daco pen (Dako, Glostrup, Denmark). Excess wash buffer was removed by tapping. Peroxidase block solution (Animal Research Kit, Daco, North America) was applied to cover the specimen, which was incubated for 10 min at 37°C and rinsed with PBS or TBS. The specimens were incubated with rabbit anti-ER (1:200), rabbit anti-PR (1:100), rabbit anti-EGF (1:100), or rabbit anti-REGF (1:500) in TBS for 40 min at 37°C. A section was prepared without the primary antibodies (replaced with PBS) to serve as the negative control. The specimens were washed with TBS and incubated at 37°C with biotin-labeled secondary antibody (Envision+System-HRP Labeled Polymer Anti-Rabbit, Daco, North America) for 40 min or mouse anti-PCNA antibody PC-10 (Dako, Glostrup, Denmark) or mouse VEGF antibody (Abcam, USA) in PBS for 20 min. Tissue sections were incubated with streptavidin-peroxidase (Animal Research Kit, Daco, North America) for 20 min at 37°C. Chromogenic reaction was conducted with DAB for 3-10 min and terminated by rinsing with water. Finally, the sections were counterstained with hematoxylin and were mounted. Yellowish-brown color was considered positive staining, and staining density was scored from 0 to +3 (0: none, +1: mild, +2: medium, and +3: severe) [14]. Five sections per sample and five fields per section were randomly selected for analysis using the Medical Image Analysis software (Motic Image plus 2.0, China).

### OS biomarker (OSB) and AO assay

Plasma samples were also obtained to analyze for OSBs (such as thiobarbituric acid reactive substance [TBARS] and a marker of lipid peroxidation) and AOs (such as superoxide dismutase [SOD] activity and total AO capacity [TAC]), as described by Schmidt *et al*. [15] and El Yew *et al*. [16]. The extent of lipid peroxidation in the plasma was assessed by measuring the TBARS by the TBARS assay kit (QuantiChrome™ a TBARS Assay Kit, Bioassay system, San Francisco Bay Area, USA). The SOD activity was determined in the plasma and tissue homogenates using the Enzychrom™ superoxide Dismutase Assay Kit (Bioassay System, San Francisco Bay Area, USA), which assesses the percentage of superoxide radicals that undergoes dismutation in a given sample [15]. The TAC was quantified by a commercially available kit (QuantiChrome™ AO Assay Kit, Bioassay System, San Francisco Bay Area, USA) that measures the oxidation of 2,29-azino-di-3-ethybenzthiazoline sulfonate by metmyoglobin, which is inhibited by non-enzymatic AOs contained in the sample.

### Statistical analysis

All results were expressed as means ± standard error of the mean and analyzed with GraphPad Prism 6.0 (GraphPad Software, San Diego, California). Analysis of variance (ANOVA) with Tukey multiple comparison *post hoc* tests was used to compare the ovarian and uterine BW ratios and lengths; thicknesses of the LE, GE, and endothelium; numbers of uterine glands; levels of hormones, OSBs, and AOs; and expression levels of steroid receptors, growth factors, and PCNA, and steroid receptors; and the concentrations of hormones, OSB, and AO. Meanwhile, two-way ANOVA with Bonferroni’s multiple comparison tests was employed to compare the BWs.

## Results

**Figure-1:**
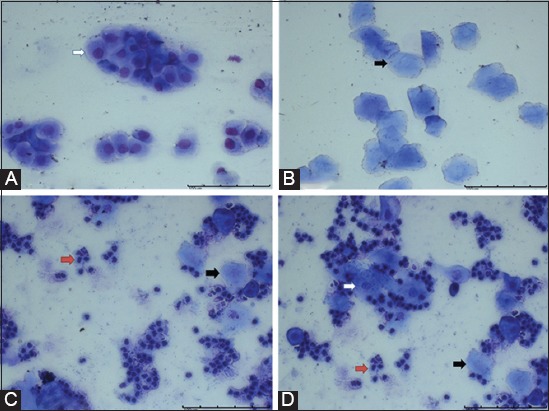
Stages of the estrous cycle in the rat as monitored by vaginal cytology. Nucleated epithelial cells (white arrow), cornified squamous epithelial cells (black arrow), and leukocytes (red arrow). (A) Pro estrus. (B) Estrus. (C) Met estrus. (D) Di estrus.

Representative photomicrographs of estrous cycle stages revealed the changes in the proportion of the vaginal cells at each stage. The rats had normal estrous cycle duration (4 days). Three cell types were observed in the vaginal smears. The relative ratios of these cells can be used to identify the estrous cycle stage of the rat on the day of sample collection. Clusters of round nucleated epithelial cells with individual lightly stained cytoplasms and oval nuclei were noted during the proestrus stage (Figure-1A, white arrow). Packed clusters of cornified squamous epithelial cells were observed during the estrus stage (Figure-1B, black arrow). Polymorphonuclear leukocytes and few cornified epithelial cells were apparent during the metestrus stage (Figure-1C). In the diestrus stage, leukocytes predominated, and the number of both cornified and nucleated epithelial cells was reduced (Figure-1D).

**Figure-2:**
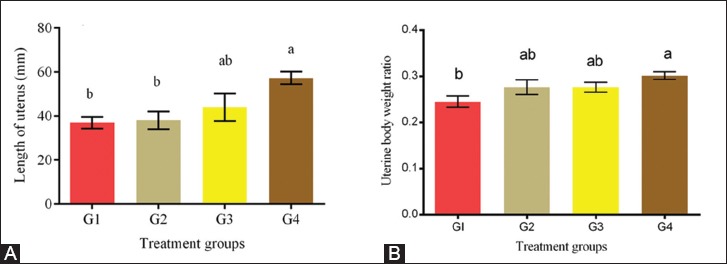
(A-B) Effect of edible bird’s nest (EBN) on uterine to body weight ratio and length change of non-pregnant rats. Data are expressed as means±standard error. Different letters a, b, and c within rows denotes significant difference at p<0.05. Notice, G1=Control; G2=EBN (30 mg/kg body weight); G3=EBN (60 mg/kg body weight); G4=EBN (120 mg/kg body weight).

**Figure-3:**
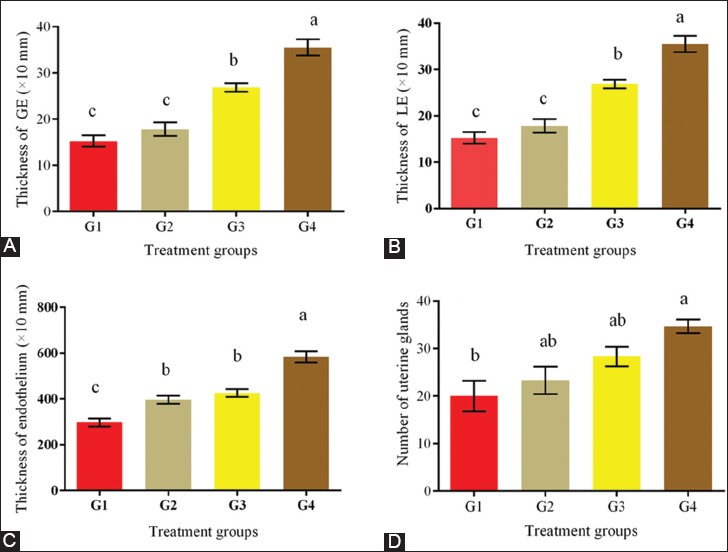
(A-D) Histomorphometric parameters evaluated in the rat uterus, measured during the proestrus phase. µm: micrometer. Data are expressed as means±standard error. Different letters a, b, and c within rows denotes significant difference at p<0.01.

**Figure-4:**
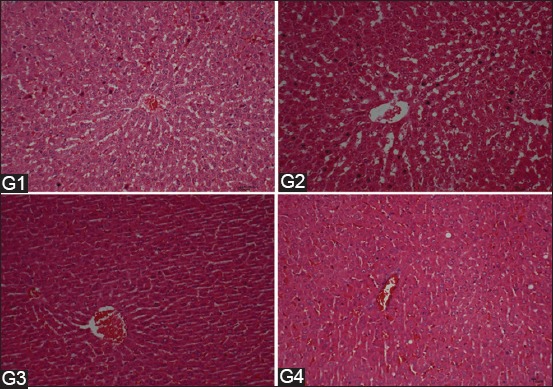
Photomicrograph reveals that histological structures of rat liver at the 8^th^ week after treatment of rats with different dosages of edible bird’s nest (EBN). G1=Control; G2=EBN (30 mg/kg body weight); G3=EBN (60 mg/kg body weight); G4=EBN (120 mg/kg body weight).

The uterine –to-BW ratios and lengths were significantly increased in the treatment groups and attained the highest value (p<0.05) in G4 (Figure-2). Uterine weights and lengths increased dose-dependently with EBN. The rat uteri were assessed in the proestrus stage. The uterine histomorphometric data from all the groups are showed in Figure-3. The thicknesses of LE, GE, and endometrium were increased dose-dependently with EBN. All treated groups achieved significantly (p<0.05) higher thicknesses of LE, GE, and endothelium than the control (Figure-3A-C). The highest number of uterine glands was observed in G4 (p<0.05) and was significantly higher than that in G1, while that in G3 was comparable to those in G2 and G4. The uterine glands and GE thickness significantly increased dose-dependently in G4 (p<0.05) with respect to that in the control. The results in G2 and G3 were insignificantly lower than that in G4 but higher than that in the control (G1). Figure-4 shows representative histological sections of the liver of the four experimental groups. The microscopic examination of the liver showed no pathological lesions were in any part of the liver in all groups.

### Expression of EGF, REGF, PCNA, and VEGF in the uterus

**Figure-5:**
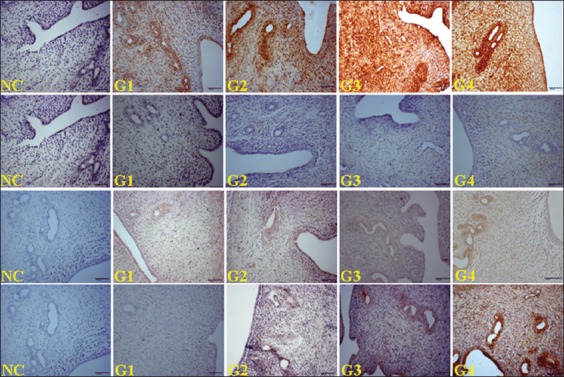
Photomicrograph sections of the uteri of rats of different experimental groups (G1, G2, G3, and G4) treated with different doses of edible bird’s nest (EBN) showing expressions of epidermal growth factor (EGF), vascular endothelial growth factor (VEGF), and proliferating cell nuclear antigen (PCNA), respectively (200×). Photomicrographs labeled with negative control (NC) represents control stains without antibody and immunity reaction for EGF (1^st^ row), REGF (2^nd^ row), VEGF (3^rd^ row), and PCNA (4^rd^ row) groups, respectively. First row shows extraordinarily higher EGF expression across groups with highest level observed at G4. For REGF, no staining signals of expression in G1 and G2. G4 had higher expression. For VEGF, remarkably higher expressions are visible in G3 and G4. Third row represents the expression of PCNA with G3 and G4 demonstrating higher expressions than the other groups.

Representative immunohistochemical photomicrographs showing the expressions of EGF, PCNA, and VEGF in the histological uterine sections of all the experimental groups are displayed in Figure-5, whereas the respective score differences are listed in Table-2. The increasing EBN dose also increased the density of stromal cells and the expression levels of EGF, REGF, PCNA, and VEGF in the uterine LE and GE. Consequently, the expression increment in all the above parameters was found significant under the highest EBN dose (G4) with respect to those in the control and other treated groups (p<0.05) (Table-2). G3 achieved the second highest expression of EGF, REGF, PCNA, and VEGF. The levels were significantly higher than those in the control and G2 (Table-2).

**Table 2:** Expressions of EGF, REGF, VEGF, and PCNA in the LE, GE, and stromal cells of uteri of rats treated with different doses of EBN and sacrificed at proestrus stage of the estrous cycle.

Parameter	Expression level (mean±standard error)			

	G1	G2	G3	G4
EGF in LE	1.5±0.3^d^	2.0±0.1^c^	2.5±0.3^b^	3.0±0.0^a^
EGF in GE	2.0±0.0^c^	2.5±0.0^b^	3.0±0.0^a^	3.0±0.0^a^
EGF in S	1.5±0.0^d^	2.0±0.0^c^	2.5±0.0^b^	3.0±0.0^a^
REGF in LE	0	0	0.5±0.0^b^	1.5±0.0^a^
REGF in GE	0	0	0.5±0.0^b^	1.5±0.0^a^
REGF in S	0	0	1.0±0.0^b^	2.5±0.0^a^
VEGF in LE	1.0±0.0^d^	1.5±0.0^c^	2.0±0.0^b^	2.5±0.0^a^
VEGF in GE	1.5±0.0^c^	1.5±0.0^c^	2.0±0.0^b^	3.0±0.0^a^
VEGF in S	1.±0.0^c^	1.5±0.0^b^	1.5±0.0^b^	2.5±0.0^a^
PCNA in LE	0	1.0±0.0^b^	2.0±0.0^b^	3.0±0.0^a^
PCNA in GE	0	1.5±0.0^c^	2.0±0.0^b^	3.0±0.0^a^
PCNA in S	0	1.0±0.0^c^	1.5±0.0^b^	2.5±0.0^a^

LE=Luminal epithelium, GE=Glandular epithelium, S=Stromal cells. Data are expressed as means±standard error. Different letters a, b, and c denotes significant difference at P<0.05. NC=Control without antibody. G1=Control, G2=EBN (30 mg/kg body weight); G3=EBN (60 mg/kg body weight); G4=EBN (120 mg/kg body weight); EGF=Epidermal growth factor, REGF=Receptor of epidermal growth factor, VEGF=Vascular endothelial growth factor, PCNA=Proliferating cell nuclear antigen, EBN=Edible bird’s nest

### Expression of steroid receptors (E_2_R and P_4_R) in uterine tissues

**Figure-6:**
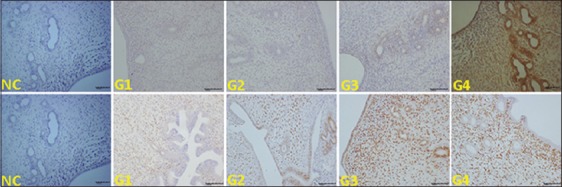
Photomicrograph sections of rat uteri of different experimental groups (G1, G2, G3, and G4). No staining for estrogen receptor was observed in G1 and G2 and show higher expression in G4. Second row shows higher expression of P_4_R in G3 and G4 (200×). Negative control (NC) represents control stain without antibody and immunity reaction.

Representative immunohistochemical photomicrographs revealing the expressions of receptors of estradiol (E_2_R) and progesterone (P_4_R) in the uterine histological sections of all the experimental groups are shown in Figure-6, whereas the respective score differences are listed in Table-3. No staining was noted for the E_2_R in the G1, G2, and G3 samples, except for the staining in the uterine gland and uterine surface epithelium in G3. By contrast, G4 attained the highest E_2_R expression (p<0.05) (Figure-6 and Table-3). The stromal cell density and the P_4_R expression in the USE and uterine gland epithelium increased with the increasing EBN dose. The rats with the highest EBN dose (G4) showed a significant increase in all the above parameters relative to those of the control and the other treated groups (p<0.05) (Table-3). G3 exhibited the second highest P_4_R expression, which was also significantly higher than those of the control and G2 (Table-3).

**Table 3:** Expressions of E_2_R and P_4_R in the LE, GE and stromal cells of uterus of rats treated with different doses of EBN and sacrificed at proestrus stage of the estrous cycle.

Parameter	Expression level (Mean±SE)				

	NC	G1	G2	G3	G4
E_2_R in LE	0	0	0	1.0±0.0^b^	3.0±0.0^a^
E_2_R in GE	0	0	0	1.0±0.0^b^	3.0±0.0^a^
E_2_R in S	0	0	0	2.5±0.0^b^	3.0±0.0^a^
P_4_R in LE	0.25±0.00^d^	1.5±0.0^d^	2.0±0.0^c^	2.5±0.0^b^	3.0±0.0^a^
P_4_R in GE	0.25±0.0^d^	1.5±0.0^d^	2.0±0.0^c^	2.5±0.0^b^	3.0±0.0^a^
P_4_R in S	0.25±0.00^d^	1.5±0.0^d^	2.0±0.0^c^	2.5±0.0^b^	3.0±0.0^a^

LE=Luminal epithelium; GE=Glandular epithelium, S=stromal cells. Data are expressed as means±SE. Different letters a, b, and c denotes significant difference at P<0.05. NC=Control without antibody. G1=Control; G2=EBN (30 mg/kg body weight); G3=EBN (60 mg/kg body weight); G4=EBN (120 mg/kg body weight). E_2_R=Nuclear estrogen, P_4_R=Nuclear progesterone, SE=Standard error, EBN=Edible bird’s nest

### OSB and AO assay

**Figure-7:**
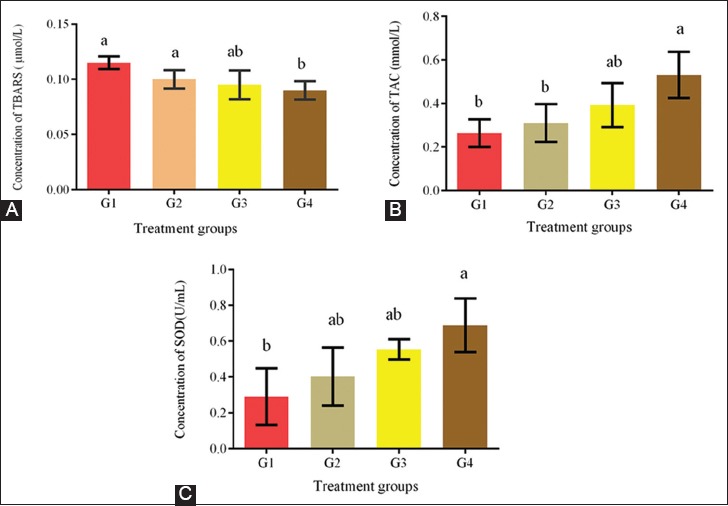
(A-C) Effect of edible bird’s nest on oxidative stress and antioxidant biomarkers in plasma. TBARS=Thiobarbituric acid reactive substance, TAC=Total antioxidant capacity, SOD=Superoxide dismutase. Data are expressed as means±standard error. Different letters a and b within rows denotes significant difference at p<0.05. G1=Control, G2=EBN (30 mg/kg body weight); G3=EBN (60 mg/kg body weight); G4=EBN (120 mg/kg body weight).

In this study, the TBARS levels, TACs, and SOD activities in the plasma from all the groups are shown in Figure-7. The decrease in TBARS level and increase in SOD activity in the treatment groups were both dose-dependent. These effects changed the redox status by enhancing the enzymatic AO defense (SOD) and increasing the TAC. These results indicated that EBN modifies and attenuates the redox system.

## Discussion

There has been an increase in interest in natural substances and their bioactive components in the past two decades. One of the primary motives was hormone replacement therapy that showed their dangerous side effects over time, whereas natural substances have generally centuries-long use without side effects [17]. According to a recent study, the estrous cycle is a regular progression affected by the release of gonadotropin-releasing hormone from the hypothalamus, gonadotropins from the pituitary gland, and sex hormones from the gonads. EBN also possesses estrogenic activity and increases the number of uterine glands [4,13]. The present results revealed that the rat BWs increased due to EBN nutritional value. According to Sen *et al*. [11], the expression of the sialic acid binding protein in human endometria is regulated by estradiol. In this study, uterine weight and length significantly increased in the treatment groups with respect to those in control. This result confirmed the correlation of the estrogen and sialic acid content of EBN to the weight and length of the uterus. In this study, histological examination of the uteri of the different treatment groups revealed EBN-induced dose-dependent changes, which were characterized by thickening of LE, GE, and endometrium and the increase in uterine gland numbers. The endometrial glands and their secretions are important in maintaining estrous cycles, conceptus survival, and growth at the peri-implantation stage [18]. Therefore, EBN enhances the utero physiological functions by maintaining uterine glandular secretions, such as growth factors, hormones, and transport proteins, and for conceptus embryogenesis [11]. In previous studies, serum toxicity markers (alkaline phosphatase, alanine transaminase, urea, and creatinine) did not change [4]. This result agrees with the lack of apparent gross pathological and histological lesions observed in any of the vital organs, including the liver in the present study. Instead, EBN protected against hydroxyl radicals that cause *in vitro* cytotoxicity on human lung cells and cellular damage from human liver carcinoma [19].

Histologically, the uterine cells and glands of rats showed increased activity when treated with EBN. Furthermore, PCNA density increased, which is a sign of DNA synthesis and cell proliferation. These results provide additional evidence to the potential role of EBN in reproduction and fertility. EGF and REGF, VEGF, and interleukine-6 play essential roles in cellular processes and are intercellular mediators that control growth, survival, and cellular differentiation, and function [20,21]. This notion agrees with our present results, where the significant expression of EGF and REGF was associated with the abundant proliferation of LE, GE, and uterine glands and the thickening of endothelium. Similar to the study of Roh *et al*. [22], the current study confirmed that EBN enhances EGF, REGF, VEGF, and PCNA expression in the GE, USE, and uterine stromal cells, thereby indicating the proliferative effect of EBN. Moreover, VEGF is an angiogenic factor in the endometrium and hence is vital to the development, maintenance, and degradation of the structure. VEGF is also a major factor for intensifying the vascularization of uterine glands and stromal cells and thus improves nutrient supply. The link between increased VEGF expression and EBN supplementation reveals how EBN promotes the proliferation of stromal cells, LE, and GE. VEGF is related to the actions of LH and angiopoietin produced in luteinizing cells [23-25]. Alterations in the hypothalamic–pituitary–gonadal axis affect the induction of steroidogenesis in the interstitial and theca cells of the ovary [26]. The present data suggested that PCNA was influenced by steroidal activity, and E_2_R and P_4_R increase PCNA production. Therefore, we deduce that EBN upregulates the PCNA expression in the uterus.

Estrogen and progesterone essentially affect the uterus through E_2_R and P_4_R [26]. Progesterone is essential for maintaining the stromal and glandular cells of the endometrium and the cells of the myometrium. This hormone also helps uphold implantation and pregnancy in all mammals. Estrogen plays an important role in the proliferation of uterine epithelia and enhances progesterone action by stimulating the PR. This study showed that the increased density of stromal cells, GE, and LE was accompanied by the significant expression of steroid receptors (E_2_R and P_4_R) associated with EBN supplementation. This result provides further evidence on the importance and action mechanism of EBN supplementation in enhancing reproduction.

In this study, EBN was apparently related to the increase in reproductive hormonal expressions of their receptors; this association points to the great effect of the EBN on rat uteri that is yet to be elucidated. Uterine E_2_R expression and uterine P_4_R expression were augmented in a dose-dependent manner and revealed the positive effect of EBN on steroid hormone production. This result agrees with that of Ismail *et al*. [4], who reported the increased serum E_2_ levels of ovariectomized rats fed on EBN dietary supplement and hence concluded that EBN can be used as estrogen therapy for ovariectomized-induced aging-related memory loss. Guzeloglu-Kayisli *et al*. [1] proposed the ovaries, along with the uterus and mammary glands, as the target organs of estrogen and progesterone.

In this study, EBN significantly lowered the TBARS levels (p<0.05) in the treatment groups as compared with those in the untreated control; however, the difference was only significant in G4. AO status was higher (p<0.05) in the treatment groups than in the control group. This finding is similar to those of a previous study, where EBN protein hydrolysates exhibit potent AO activity that lowers OSB levels [19,27].

## Conclusion

The present findings suggested that EBN supplementation could be used to enhance reproduction and uterine functions by increasing the proliferation of uterine structures, such as LE, GE, and stromal cells. EBN can also enhance AO activity, and upregulate the expression of EGF, REGF, and VEGF, PCNA, and steroid receptors on uteri.

## Authors’ Contributions

This study was intellectualized by AAA, NY, MZAZ, AWH, RY, MAA, and BHA. The investigations were performed by AAA, MAA, and BHA under the supervision of NY, MZAZ, AWH, and RY. Statistical analysis was carried out by AAA and NY. Original manuscript was written by AAA, reviews and editing werefinalized by AAA, NY, MZAZ, AWH, RY, MAA, and BHA. The final manuscript was read and approved by all authors.
